# Differential amino acid usage leads to ubiquitous edge effect in proteomes across domains of life that can be explained by amino acid secondary structure propensities

**DOI:** 10.1038/s41598-024-77319-4

**Published:** 2024-10-26

**Authors:** Juliano Morimoto, Zuzanna Pietras

**Affiliations:** 1 School of Natural and Computing Sciences, Institute of Mathematics, University of Aberdeen, Fraser Noble Building, Aberdeen, AB24 3UE UK; 2 Programa de Pós-graduação em Ecologia e Conservação, Universidade Federal do Paraná, Curitiba, 82590-300 Brazil; 3 Department of Physics, Chemistry and Biology (IFM), Linköping University, Linköping, Sweden; 4 Present Address: Wissenschafskolleg zu Berlin, 10 Wallotstraße, Berlin, Germany

**Keywords:** Genetic code, Structural biology, Environmental responses, Physiology, Proteins, Molecular modelling, Molecular biology, Genome informatics, Proteome informatics

## Abstract

Amino acids are the building blocks of proteins and enzymes which are essential for life. Understanding amino acid usage offers insights into protein function and molecular mechanisms underlying life histories. However, genome-wide patterns of amino acid usage across domains of life remain poorly understood. Here, we analysed the proteomes of 5590 species across four domains and found that only a few amino acids are consistently the most and least used. This differential usage results in lower amino acid usage diversity at the most and least frequent ranks, creating a ubiquitous inverted U-shape pattern of amino acid diversity and rank which we call an ‘edge effect’ across proteomes and domains of life. This effect likely stems from protein secondary structural constraints, not the evolutionary chronology of amino acid incorporation into the genetic code, highlighting the functional rather than evolutionary influences on amino acid usage. We also tested other contemporary hypotheses regarding amino acid usage in proteomes and found that amino acid usage varies across life’s domains and is only weakly influenced by growth temperature. Our findings reveal a novel and pervasive amino acid usage pattern across genomes with the potential to help us probe deep evolutionary relationships and advance synthetic biology.

## Introduction

Proteins perform a wide variety of vital roles which depend on their structure and ultimately, their amino acid composition^1,2^. The frequency of, or changes to, amino acid profiles in the proteome provide insights into evolutionary mechanisms shaping genomes and their products^3–8^ and can be used in disease diagnostics^9,10^ and synthetic biology^11–13^. Despite this, we still lack a proper understanding of amino acid usage and frequency across domains of life, and how proteomes change as a result of the environment and intrinsic physiological constrains imposed by evolving organisms.

Two competing hypotheses exist regarding similarities and differences in amino acid usage in proteomes among species. On the one hand, previous studies have shown that amino acid profiles across proteomes differ among domains of life primarily due to lifestyle^4,5,14^. On the other hand, more recent studies suggested that proteomes are conserved among species from different domains of life and contain lineage-specific information on the amino acid requirements to improve fitness^15,16^. This hypothesis is striking because only specific protein sites (e.g. secondary structures in catalytic motifs) are highly conserved, with the remaining of the protein sequence evolving under fewer biophysical and structural constrains^6,7,17^. Moreover, not all proteins are highly expressed which is known to slow down protein sequence evolution and increase conservation of sequences^18,19^. Nonetheless, amino acid usage appears remarkably consistent^15,16^. A possible justification for why amino acid profiles might be similar across domains of life is that amino acid profiles are shaped by energetic and biophysical constrains and the proteome reflects the overall cost minimization of amino acid synthesis, scavenging opportunities and protein production^3,20–22^. Conflicting evidence for the conservation of amino acid profiles in proteomes across domains of life exist^4,8,14,15,20,23,24^, but data is taxonomically limited preventing a proper test of the generality of the hypothesis. Furthermore, other investigations that reveal new patterns in amino acid usage have not been conducted, rendering our understanding of proteome patterns incomplete.

Here, we collated a comprehensive dataset of 5590 proteomes of species from across four domains of life (bacteria, eukaryote, viruses, and archaea) to test whether amino acid usage profiles differ or not among distant groups. Next, we incorporated optimal growth temperature for 296 of these proteomes to test whether differences in amino acid usage was indeed a product of the interaction with the environment. We then explored our database to uncover novel patterns in amino acid usage across proteomes. In particular, we tested whether amino acid usage is shaped amino acid rank order and if these rank order based on amino acid usage correlate with the evolutionary origins of amino acids in living organisms. Our findings advance our understanding of proteomes and open new avenues of research on the patterns underlying amino acid usage across domains of life.

## Results and discussion

### Proteomes differ among distant domains of life

**Fig. 1:**
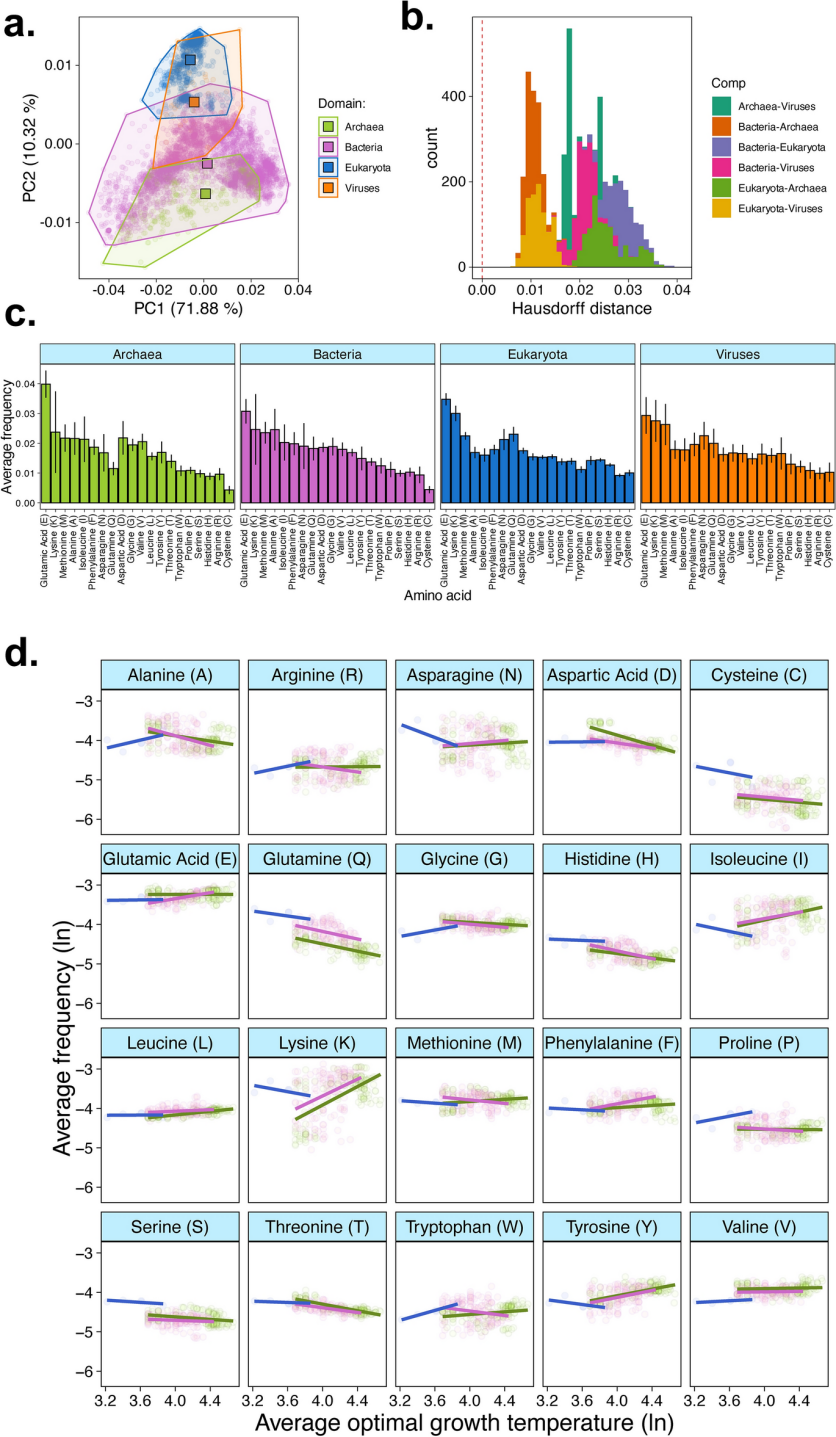
Amino acid frequencies across proteomes and the effects of growth temperature. (**a**) Principal component analysis (PCA) on standardised amino acid frequencies reveals that amino acid usage across genomes in the four domains of life differ. Standardisation was done by dividing the amino acid frequency by the number of redundant codons (see “Materials and methods”). Dots represent the centroids for each of the clusters (domains of life). (**b**) Sampling distribution of Hausdorff distances comparing the PCA clusters. Values for the Hausdorff distances equating zero represent no differences between two sets of points (see “Materials and methods” for details). (**c**) Average standardised amino acid frequencies in proteomes across four domains of life. (**d**) The relationship between standardised amino acid frequencies and optimal growth temperature reveals that environment only has a minor effect on amino acid profiles. In panel (**d**), axes were ln-transformed. Note that (**a**–**c**) tests the lineage-specific hypothesis whereas (**d**) tests the lifestyle hypothesis (see Main Text).

Two competing hypotheses suggest that amino acid usage in proteomes differ or not among distant groups of living organisms. The lineage-specific hypothesis suggests that amino acid frequencies across proteomes are relatively constant even for distantly related species. On the other hand, the lifestyle hypothesis suggests that environment conditions, such as optimal growth temperature, are the primary drivers of amino acid usage differences. To test these, we first analysed the proteome of 5590 species with complete annotated genomes and identified taxonomy from the NCBI database (see Supplementary Materials for Materials and Methods and Extended Data 1). For each species, we calculated the amino acid profile as the sum of individual amino acid frequencies divided by the total sum of amino acid counts as in^15^, which resulted in amino acid profiles for 328 Archaea species, 4107 Bacteria species, 1118 Eukaryotes and 37 Viruses. In our data (F_1,111792_ = 7247.30, p < 0.001, Table S1), as in previous studies^3^, the number of redundant codons and the frequency for the corresponding amino acid were positively correlated and to account for this, we used standardised amino acid frequency (i.e. amino acid frequency divided by the number of redundant codons). We found no evidence that the amino acid frequencies were conserved across domains of life as shown by both differences in the principal component analysis (PCA) clusters (Fig. 1a, b) and in the average amino acid usage frequency across domains (Domain*Amino acid frequency: F_3,111720_ = 398.9, p < 0.001, Fig. 1c). This was confirmed using a PERMANOVA on the amino acid usage profile across domains of life (PERMANOVA: F_3,5586_: 328.27, p = 0.004). This effect was driven by proteome-wide differences in amino acid frequencies and not by a single or few amino acids having disproportionate effect, as shown in our PCA analysis (Fig. 1a) and the average amino acid usage frequencies across domains (Fig. 1c). Nonetheless, it is worth highlighting the nearly two-fold increase in frequency of cysteine (C) in eukaryotes and viruses compared with prokaryotes which is consistent with the literature^25^. The differences in amino acid profiles across proteomes were observed when we analysed proteomes without codon standardisation (Supplementary file 1; F_3,111720_ = 419.57, p < 0.001) as well as for proteomes standardised using amino acid molecular weight, which is known to correlate negatively with amino acid frequency^24^ (Supplementary file 1; Domain*Amino acid: F_3,111720_ = 452.12, p < 0.001). These results show that amino acid profiles across proteomes are not conserved in species from distant domains of life.

### Environment weakly explains variation in proteomes

Past studies hypothesised that variation in amino acid profiles were driven by environmental conditions such as optimum growth temperature^5,14,15,26^ (the lifestyle hypothesis). Thermophilic proteomes were shown to be under stringent evolutionary constraints^27^ which affect amino acid profiles relative to mesophilic proteomes^4,26^ and can lead to highly contrasting amino acid usage profiles^4^. However, these studies were taxonomically biased because they lacked representation of large sets of mesophilic archaea^4,14,26^. We incorporated to our proteome dataset information on optimal growth temperature (in ^o^C) for 296 species of Eukaryotes (*n* = 5), Bacteria (*n* = 149) and Achaea (*n* = 141) obtained from the ThermoBase database^28^ and the supplementary data in^24^ (Extended Data 2). From those, 65.8% (n = 195) were mesophilic (optimal growth up to 70 °C) whereas the remaining 34.2% (n = 101) were thermophilic (optimal growth between 70 and 110 °C). We firstly measured how much additional variance was explained when temperature was added as covariate in the model of amino acid frequencies for the 296 species for which growth temperature was available. Temperature had a statistically significant but small contribution to explaining the variance in our model (Likelihood ratio: $\chi$^2^-value: 1113.2, p < 0.001; *R*^*2*^ with vs without growth temperature: 0.831 vs 0.797), suggesting that the effects of growth temperature on amino acid profiles were small. One explanation for this is that only few amino acids which are thermolabile respond negatively to growth temperature and thus, the potential for variation at the proteome level is masked by other amino acids. These results do not contradict previous comparisons of proteomes using pairwise or sophisticated data transformations^4,14,26^ but they show that the magnitude of the influence of the environment on the proteome is minor.

Next, we investigated the strength of the relationship between amino acids and growth temperature to disentangle which amino acids, if any, were linked to increasing growth temperatures. For instance, cysteine has been considered an environment-sensing amino acid and is also thermolabile^29^. Our data showed that amino acid frequencies differed with increasing growth temperature among domains of life (Growth Temperature*Domain*Amino acid: F_38,5800_ = 3.005, p < 0.001; Fig. 1d). This was driven primarily by the an increase in frequency of Alanine (A) and Arginine (R) alongside a strong decrease in frequency of Asparagine (N) and Lysine (K) with increasing growth temperature in eukaryotes but not in archaea or bacteria, a decrease in frequency of Aspartic acid (D) with increasing growth temperature in archaea but not in bacteria and eukaryotes, and an increase in the relative frequency of Glutamic acid (E) and Phenylalanine (F) with increasing growth temperature in bacteria but not archaea or eukaryotes (Fig. 1d). There were also statistically significant main effects (Amino acid: F_19,5800_ = 5.526, p < 0.001, Table S1) and two-way interactions (Growth temperature*Amino acid: F_19,5800_ = 2.384, p < 0.001; Domain*Amino acid: F_38,5800_ = 3.280, p < 0.001, Table S1) on amino acid frequency. These results confirmed a previous report^26^ that increasing growth temperature leads to an overall decrease in frequency of the thermolabile amino acids such as cysteine (C) and glutamine (Q)^5,30,31^, a pattern which we observed in our data in eukaryotes, archaea and bacteria. Cysteine has also been considered an anomalous amino acid due to lower frequencies than expected by cost models across proteomes^14,24,30^ although these studies did not directly control for growth temperature which could explain the relatively lower cysteine frequency than expected. Nevertheless, our results show that despite its low frequency, thermolabile amino acids in the proteome negatively correlate with higher optimal growth temperatures. More broadly, our results show that environmental effects in proteomes are minor.

### Few amino acids predominantly appear in first and last ranks

**Fig. 2:**
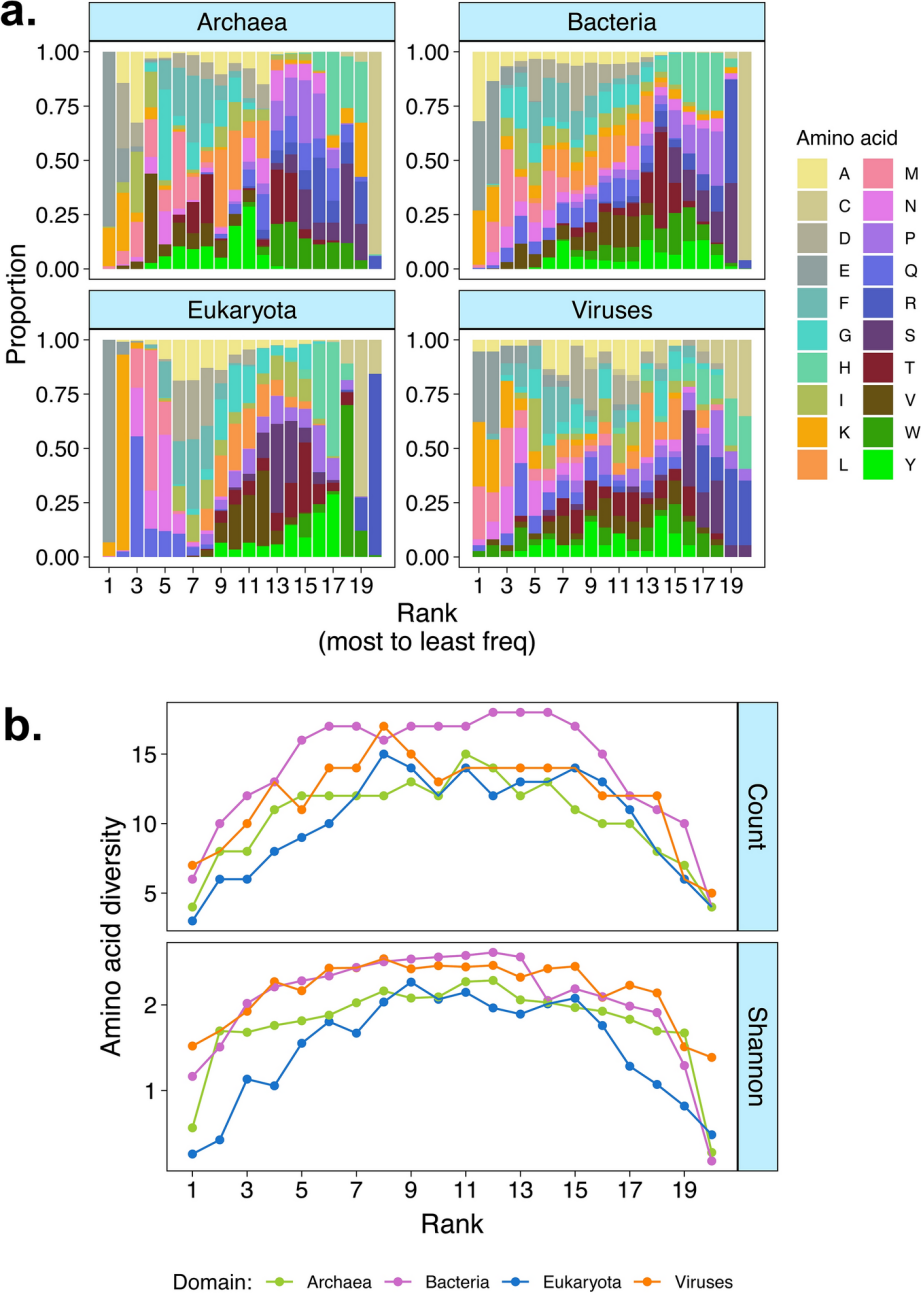
Amino acid diversity decreases in high and low usage ranks. (**a**) Amino acid proportions by rank across domains of life. (**b**) Amino acid diversity calculated as the Shannon–Wiener index (see “Materials and methods”) by rank, showing that diversity decreases at the higher and lower ranks. This means that only few amino acids are frequently or rarely used, while almost all amino acids can be seen at intermediate ranks.

We then ranked amino acids from most to least frequently used in proteomes to investigate their usage frequencies by rank. Our data shows that the proportion of amino acid by rank were similar across domains of life (Domain*Rank*Amino acid: F_57,771_ = 1.097, p = 0.294), supporting the assumption that amino acid usage by rank is shaped by cost-minimization constrains across domains of life. Our data also showed that amino acids were not used uniformly across ranks (Rank*Amino acid: F_19,888_ = 9.376, p < 0.001; Fig. 2a). This suggested that some amino acids might be differentially prevalent (or even altogether absent) across ranks, which could highlight patterns of amino acid preferential use or avoidance.

Although we did not measure amino acid usage costs directly, our rationale was that amino acid usage by rank could reflect the costs of amino acid usage assuming cost-minimization^32^. In this context, we predicted that (a) few amino acids are physiologically cheap and/or have been incorporated first into the genome to be highly abundant, (b) many amino acids have intermediate frequencies and (c) few amino acids are physiologically expensive and/or have been newly incorporated into genomes, thus having relatively low frequencies. If this pattern is consistent across taxa and domains of life, this would generate an inverted U-shape curve when we measure the diversity of amino acids that are used relative to their rank frequencies, with few amino acids in the most and least frequent ranks and many amino acids at intermediate ranks. Thus, under cost-minimization, only few amino acids are expected to be most or least frequently used, leading to a non-linear relationship between amino acid diversity and frequency ranks. This lower diversity at the edges of the amino acid frequency ranks resemble similar edge effects found in ecology^33,34^. We therefore tested whether amino acid usage by rank displayed such edge effect in proteomes across domains of life. To test this, we analysed the diversity of amino acids within each rank to assess how many amino acids (raw counts) and their weighted proportions (Shannon–Wiener diversity index) were present across ranks (see Eq. 1 in “Materials and methods”). Our data showed that amino acid diversity by rank varied linearly and non-linearly with rank across domains in both raw counts (Rank*Domain: F_3,68_ = 3.962, p = 0.011; Rank^2^*Domain: F_3,68_ = 3.408, p = 0.022, Table S1) and Shannon diversity index (Rank*Domain: F_3,68_ = 3.092, p = 0.032; Rank^2^*Domain: F_3,68_ = 6.438, p < 0.001). It also confirmed the strong non-linearity of amino acid usage by rank (Counts: Rank^2^: F_1,68_ = 578.117, p < 0.001; Shannon: Rank^2^: F_1,68_ = 425.21, p < 0.001, Table S1) which was not observed for the linear term (Counts: Rank: F_1,68_ = 0.001, p = 0.967; Shannon: Rank: F_1,68_ = 0.987, p = 0.323). These results corroborate our predictions and highlight a novel edge effect where the diversity of amino acids that appeared in ranks 1–2 and 19–20 was lower than the diversity of amino acids in intermediate ranks, an effect observed across all domains of life (Fig. 2b). It is unlikely that the edge effect was a statistical artifact because it was observed when the data was analysed without codon standardization or with standardization by amino acid molecular weight (Supplementary File 1) and for proteomes from increasing growth environment (Supplementary File 2).

### The edge effect is present in the amino acid profiles of secondary structures

**Fig. 3:**
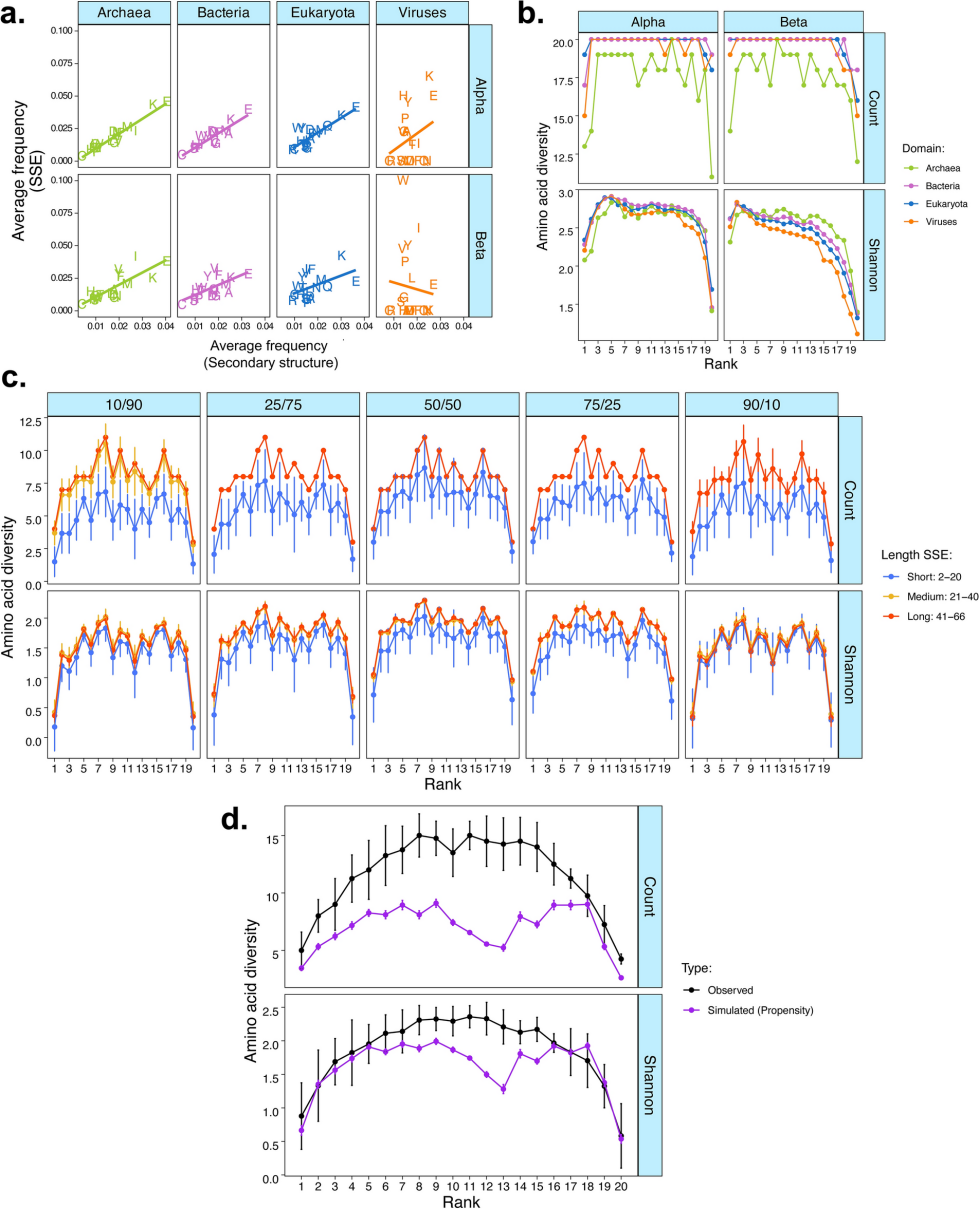
Edge effect is likely driven by conformational properties of amino acids. (**a**) The relationship between average standardised amino acid frequency in the proteome (y-axis) and on the secondary structures (x-axis). (**b)** Amino acid diversity by rank, calculated using the Shannon–Wiener index, within secondary structures. (**c**) Amino acid diversity by rank, calculated using the Shannon–Wiener index, of simulated proteins with varying mixtures of α-helices/β-strands ratio. Note that medium and long SSE length tend to overlap. (**d**) Comparison between amino acid diversity by rank calculated using the Shannon–Wiener index from the proteome analysis (Observed) and from simulated data using propensity to form secondary structure from37 [Simulated (Propensity)].

The edge effect appears to be ubiquitous and thus, we hypothesised that its cause must also be rooted into fundamental biophysical principles shaping amino acid usage. It is well established that secondary structures such as $\alpha$-helices and $\beta$-strands are often conserved among protein superfamilies even in distantly related species ^35–38^. Moreover, amino acids differ in their propensity to form $\alpha$-helices and $\beta$-strands^39,40^ which could influence how often they are used in proteomes, depending on their role in secondary structures. Thus, we hypothesised that amino acid frequencies in the proteome reflected their propensity to appear in protein secondary structures, such as $\alpha$-helices and $\beta$-strands, which could explain the edge effect and why few amino acids appear in most and least frequent ranks in proteomes in all domains of life. To test this, we analysed the amino acid frequency in secondary structures of 40,885 PDB unique entries from 3512 species across all four domains of life, selected from a subset of structures with low sequence similarity and solved at high-resolution (< 3 Å) from the PISCES culling database^41,42^. We first tested whether the average amino acid frequency in the proteome correlated with the average frequency of the amino acid in $\alpha$-helices and $\beta$-sheets and found that the average frequency in the proteome and secondary structures were statistically correlated in $\alpha$-helices (Frequency SSE: F_1,72_ = 92.08, p < 0.001) and $\beta$-sheets (Frequency SSE: F_1,72_ = 8.846, p = 0.003) but differed across domains of life for both secondary structure types (Domain*Frequency SSE $\alpha$_:_ F_3,72_ = 28.279, p < 0.001; Domain*Frequency SSE $\beta$_:_ F_3,72_ = 11.870, p < 0.001, Table S1). This was driven by a weaker positive relationship between amino acid frequencies in the proteome and $\alpha$-helices and a negative relationship between amino acid frequencies in the proteome and $\beta$-sheets in Viruses compared with other domains of life (Fig. 3a).

Next, we hypothesised that the proteome-level edge effect could be an emerging property of the edge effects in secondary structures. We tested this by first ranking amino acids from most to least frequently used in either $\alpha$-helices or $\beta$-strands for each species and measured the diversity of amino acids in each rank across the domains of life. There was evidence of a non-linear edge effect in both $\alpha$-helices and $\beta$-strands (Rank^2^: F_1,144_ = 184.833, p < 0.001) but this varied depending on secondary structure and domain. For instance, the edge effects on $\beta$-strands were relatively stronger in higher ranks as opposed to lower ranks, while the edge effects on $\alpha$-helices were more relatively symmetric showing a more characteristic inverted U-shape curve (Rank^2*^SSE: F_1,144_ = 21.719, p < 0.001; Fig. 3b). The non-linearity pattern of the rank-frequency curve for $\beta$-strands was less accentuated in viruses which drove a weak but statistically significant interaction between domain and the non-linear effect of rank (Rank^2*^Domain: F_3,144_ = 2.779, p = 0.043; Fig. 3b, Table S1). These results show that the edge effect observed at the proteome-level was also present in the frequency rank of secondary structures, suggesting that the edge effect at the proteome level could be an emerging property of how amino acids form secondary structures.

### The edge effect on amino acid diversity emerges from amino acid conformational properties

Our results for amino acid diversity in secondary structures resembled the distribution of amino acid propensities to form $\alpha$-helices or $\beta$-strands reported in the classical work by Chou and Fasman^39^, where distributions of amino acid propensities for $\alpha$-helices were relatively symmetric while the propensity for $\beta$-strands were rightly skewed (Supplementary File 3). This led us to hypothesise that amino acid secondary structure propensities could be the biophysical constrain that gives rise to the edge effect at the proteome level, because amino acids could be differentially selected and used based on their secondary structure propensities. To test whether secondary structure propensity could alone replicate our findings, we simulated 54,400 sequencies of $\alpha$-helices and $\beta$-strands of varying lengths (from 6 to 66 in increments of 8 residues) where amino acids composition of these simulated secondary structures were selected with probability based only on their propensity to form $\alpha$-helices and $\beta$-strands as in^39^. This gave us a pool of $\alpha$-helices and $\beta$-strands with varying amino acid profiles which were representative of their secondary structure propensities. From this pool of simulated secondary structures, we randomly sampled $\alpha$-helices and $\beta$-strands to assemble 153,450 virtual proteins containing a mixture of these secondary structures. We simulated virtual proteins that were small (2–20 secondary structures), medium (21–40 secondary structures) or large (41–66 secondary structures), each of these with a mixture of secondary structures ranging from proteins that were primarily made of $\alpha$-helices (90–10%), balanced (50–50%) or $\beta$-strands (90–10%). As expected, the mixture of secondary structures (F_4,8970_ = 178.066, p < 0.001) and length (F_2,8970_ = 162.58, p < 0.001) of the simulated proteins influenced amino acid rank diversity. However, there was strong evidence that the edge effect could be rescued (Rank^2^: F_1,8970_ = 258.30, p < 0.001; Fig. 3c) independently of length and mixture (Length* Rank^2^: F_2,8970_ = 0.069, p = 0.932; Mixture* Rank^2^: F_4,8970_ = 0.790, p = 0.531; Fig. 3c, Table S1). The edge effect disappeared in simulations where amino acids were drawn with equal probabilities (Supplementary File 4 and Supplementary File 5), supporting that the edge effect is an emerging property of amino acid-specific secondary structure propensities.

We then tested how the simulations compared to our observed data in relation to the edge effect. To do this, we compared the rank-frequency curve from the proteomes in our data base with the curve obtained from the simulations using propensity to form secondary structures from literature^39^. We recapitulated the same edge effect observed in our proteome dataset with our simulation parameterised solely with amino acid secondary structure propensity as in ^39^. Both amino acid diversity (Rank^2^*Data type (simulated vs observed): F_1,36_ = 0.172, p = 0.680) and amino acid count showed evidence of edge effects comparable to our observed data (Rank^2^*Data type (simulated vs observed): F_1,54_ = 0.454, p = 0.504; Fig. 3d). Simulations had lower raw amino acid counts per rank (F_1,36_ = 28.963, p < 0.001, Table S1) although not lower diversity (F_1,36_ = 3.135, p = 0.085; Fig. 3d). These results confirm that amino acid secondary structure propensities could underpin the edge effect on amino acid rank diversity.

### Amino acid usage rank is independent of their evolutionary origin

**Fig. 4:**
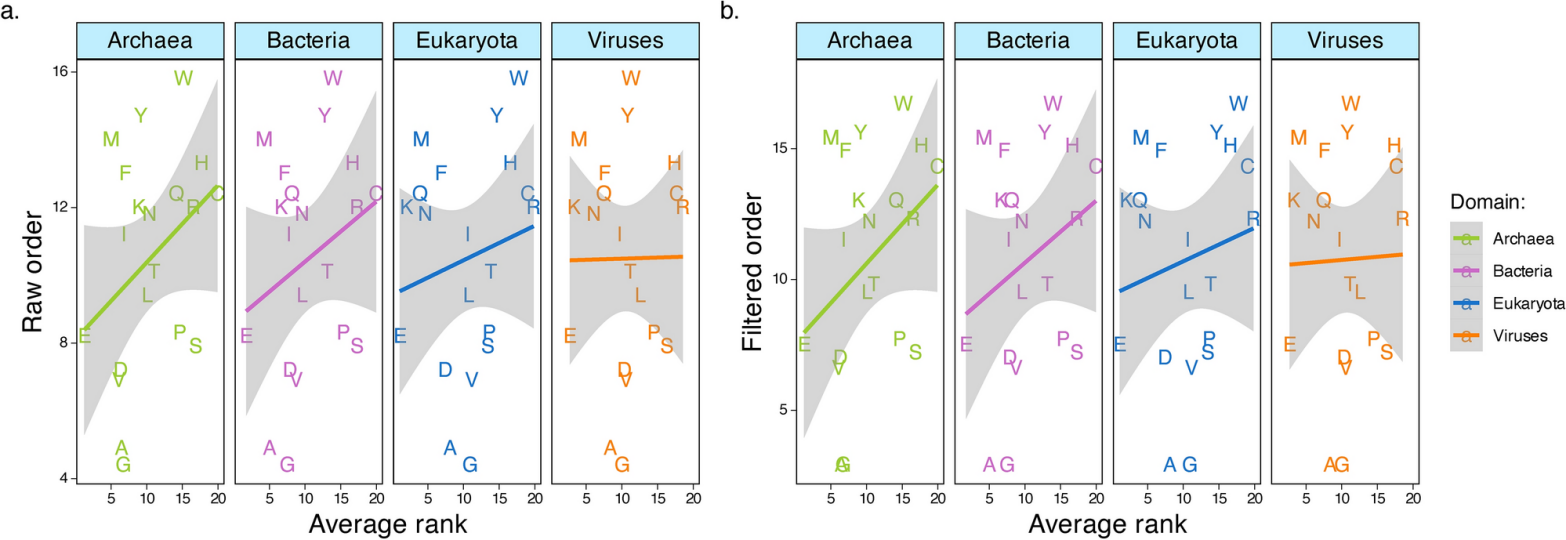
Relationship between average rank from amino acid usage and average rank from evolutionary chronology as in Trifonov^43^. (**a**) ‘Raw order’ means unfiltered average chronology ranks from the 40 criteria and (**b**) ‘Filtered order’ means the filtered average chronology ranks, accounting for correlation between the 40 selection criteria as in Trifonov^43^. 95% confidence intervals on the slopes shows that none of the slopes are statistically significant (Table S1).

The evolutionary origin of amino acids into the proteome could influence the frequency in which they are used and therefore, contribute to the edge effect. Two consensus sequencies of amino acid evolutionary chronology exists^43^ and we tested whether the average rank of amino acids based on their usage matched their average rank based on their evolutionary chronology. There was no evidence that average amino acid rank from frequency correlated with average rank from evolutionary chronology across domains of life for neither (Raw: F_3,72_ = 0.405, p = 0.749; Filtered: F_3,72_ = 0.390, p = 0.759; Fig. 4, Table S1). These results show that amino acid rank usage is determined by functional constrains on their use above evolutionary chronology. It is possible that the relationship between amino acid evolutionary origin in the genetic code and average rank is present for ancestral coding genes but not for genes that evolved more recently^44^. This could mask the relationship between evolutionary chronology and average amino acid rank computed here. Future studies which incorporate the evolutionary history of individual genes will help elucidate this. Nonetheless, our results suggest that the edge effect at the proteome level is unlikely to be driven by asymmetries in amino acid evolutionary chronology.

## Conclusion

We showed that a few amino acids are most and least frequently used in proteomes across domains of life and that this effect was driven by amino acid secondary structure propensities. This has implications to our understanding of the dynamics underpinning protein structure evolution and to our estimates of amino acid substitution rate in matrices used for amino acid sequence alignments to probe deep evolutionary history^45^. This is because the edge effect, which was pervasive across domains of life, indicates that few amino acids might be consistently more, or less, frequent in the proteome which may influence the substitution rates estimated in these matrices. We also showed that, despite the exceptions related to the edge effect, the overall amino acid usage profile of the proteomes varied among domains contradicting recent hypotheses^15,16^. Our results also showed that environmental conditions as measured by optimal growth temperature had a significant but minor effect on the amino acid frequency, including of thermolabile amino acids. Previous studies predicted a larger effect of optimal growth temperature on proteomes^4^. This discrepancy is likely because our dataset has sampled a higher proportion of mesophilic species, providing a better resolution for the effects of optimal growth temperature on amino acid frequency across proteomes on a continuous scale. Collectively, our findings give new insights into the relationship between structure and function of amino acid and proteins, highlighting a novel universal constrain shaping amino acid usage across proteomes. Proteomes contain critical knowledge about genome and protein evolution^4,5^ and can inform new ways to probe deep evolutionary histories between proteins with potential applications to protein engineering strategies in the era of synthetic biology^46,47^.

## Materials and methods

### Amino acid profiles and growth temperature

All analyses and simulations were conducted in R 4.3.2^48^. NCBI data was accessed and retrieved on or before January 2nd 2024. Translated coding sequences were downloaded from FTP servers and fasta files were processed in the statistical software R version 4.3.2^48^ to estimate amino acid profiles. The list of specie and their NCBI accession number is given in Extended Data 1. Only reference sequence (RefSeq) genomes were considered to ensure maximum coverage and annotation accuracy. For consistency of analysis across domains, we included proteomes from hosts without intracellular organelles. Because of the positive correlation between the number of redundant codons and the frequency of amino acids, we standardised our amino acid profiles, dividing amino acid frequency by the number of redundant codons. To confirm that our findings, particularly related to the edge effect, were not due to the standardization, we also analysed our amino acid profile data using no-standardization (Supplementary File 1) or standardization by amino acid molecular weight (Supplementary File 2). After standardization by codon, there was no statistical relationship between the natural log-frequency and natural log-ATP costs controlled by codon redundancies based on the amino acid costs reported in^20^ (F_1,72_ = 1.349, p = 0.249, Supplementary File5). Average optimum growth temperature was retrieved from the ThermoBase database^28^ and consolidates with supplementary data for eukaryotes from the supplementary material in^24^. ThermoBase was accessed on March 1st 2024. The final dataset of 296 species of which 142 Archaea and 149 Bacteria and 5 Eukaryotes (Extended Data 2) were used. We validated our calculations of amino acid frequencies using an independent dataset (i.e. RACCOON dataset^49^) which contained 26 species that were also present in our dataset. There were no statistically significant differences between the estimates of amino acid frequency between our dataset and the RACCOON dataset for the 26 species (*t*-value = 1.295, df = 519, p = 0.196) and the edge effect found here was also observed in the RACCOON dataset (Supplementary File 5). For our analysis of amino acid frequency in secondary structures, we downloaded the PDB structural information using the ‘bio3d’ package^50^ of 40,885 PDB unique entries from 3512 species across all four domains of life, selected from a subset of the PISCES PDB culling database^41,42^; PDB entry IDs are given in the Extended Data 3.

### Statistical analysis

Plots were made using the ‘ggplot2’ package^51^ and mixed linear regressions were fitted using the ‘lme4’ and ‘lmerTest’ packages^52,53^. To test if amino acid profiles differed across domains of life, we adopted two approaches. First, we ran a principal component analysis (PCA) using the ‘prcomp’ inbuilt R function which revealed the amino acid usage profile of each domain of life. To ascertain whether these profiles were different, we compared the clusters using a bootstrapping approach with 1000 replicates and calculating the Hausdorff distance between bootstrapping samples. Hausdorff distances applied to biological data is explained in details here^54^ but briefly, Hausdorff distance is a distance metric used to compare two sets. When the distance value is zero, the two sets are identical. For inference purposes, we assumed that, since all pairwise comparisons resulted in Hausdorff distance greater than zero, the amino acid profiles shown in the PCA analysis were different. We confirmed differences in codon-standardised amino acid usage profiles using a PERMANOVA test through the ‘adonis2()’ function of the ‘vegan’ package with 200 permutations and default parameters. We also fitted a linear mixed model using amino acid frequency as dependent variable, amino acid type and domain of life (and their interaction) as fixed effects, and species as random effects to account for multiple measurements of amino acids for each species. These two approaches revealed similar patterns. A similar mixed model was fitted to analyse the effects of growth temperature but incorporating average optimal growth temperature and the three-way interactions with amino acid type and domain of life. In all models, amino acid frequency was log-transformed to improve fit of the data. Likelihood ratio test to evaluate the increase in explanatory power by adding growth temperature was done by fitting two models, with and without growth temperature, and comparing using the ‘anova’ function of the ‘stats’ package^55^, as well as the ‘r-squaredGLMM’ function of the ‘MuMIn’ package^56^ to obtain conditional R^2^ for our mixed models. Proportion of curve classes within each domain of life was done using the ‘chisq.test’ function of the ‘stats’ package. We fitted a binomial generalized linear model with *quasi* errors using the ‘glm’ function to analyse the proportion of amino acid per rank as response variable and the three-way interactions between rank, amino acid, and domain of life as independent variables. For analysis of the relationship between amino acid average frequency in the proteome and on secondary structures, we used a general linear model with average frequency in the proteome as response variable and the interaction between average amino acid frequencies in the secondary structures and domain as independent variables. We estimated amino acid diversity per rank as the count of different amino acids per rank as well as the Shannon–Wiener diversity index per rank using the ‘tabula’ package^57^ which estimates the index using the following equation:

$$H^{\prime} = - \mathop \sum \limits_{i = 1}^{S} \frac{{n_{i} }}{N}*\ln \left( {\frac{{n_{i} }}{N}} \right)$$

where *S* represents the total number of amino acids *i* by rank and *ln* is the natural logarithm. $\frac{{n_{i} }}{N}$ represents the proportion of each amino acid *i* by rank^57^ (see also^58^). Because we were interested in edge effects, which are non-linear (i.e. inverted U-shape), we fitted general linear models using either diversity metric as dependent variable, the non-linear effects of rank and its interaction with domain for observed data or the non-linear effects of rank and their interaction with sequence length, mixture of secondary structures, and their three-way interactions for our simulations. To compare whether the edge effect was rescued in our simulations, we fitted a general linear model with either diversity metric as dependent variable, the non-linear effects of rank and its interaction with data type (i.e. simulation from propensity (“Propensity”, simulation using PDB data (“Data”) and observed proteome-level data; Fig. 3d). We also regressed average rank estimated by two methods of evolutionary origins of amino acids in the genetic code from ^43^ with the weighted average rank of each amino acid from our proteome analysis. A table with the complete output of all models presented in this paper is given in Supplementary Table 1.

## Supplementary Information

Supplementary Information 1.

Supplementary Information 2.

Supplementary Information 3.

Supplementary Information 4.

Supplementary Information 5.

## Data Availability

All data are provided as supplementary material. Raw proteome data and code for the analysis and figures is provided in the GitHub repository: https://github.com/jmor2753/Morimoto-Pietras-2024-Sci-Rep.git.
