# Supplementary material for: Differential amino acid usage leads to ubiquitous edge effect in proteomes across domains of life that can be explained by amino acid secondary structure propensities
Source: Sci Rep. 2024 Oct 26;14:25544. doi: 10.1038/s41598-024-77319-4 (PMC11513089; doi:10.1038/s41598-024-77319-4)
Supplement: Supplementary file 5 — Supplementary Information 5. [file 41598_2024_77319_MOESM5_ESM.pdf]

Supplementary Material: "Differential amino acid usage leads to ubiquitous edge effect in proteomes across domains of life that can be explained by amino acid secondary structure propensities"

Author list: Juliano Morimoto[1,2*], Zuzanna Pietras[3]

Authors' Affiliations:

[1] Institute of Mathematics, School of Natural and Computing Sciences, University of Aberdeen, Fraser Noble Building, Aberdeen, UK AB24 3UE

[2] Programa de Pós-graduação em Ecologia e Conservação, Universidade Federal do Paraná, Curitiba, 82590-300, Brazil

[3] Department of Physics, Chemistry and Biology (IFM), Linköping University, Sweden

Contact information

Dr Juliano Morimoto

Institute of Mathematics, University of Aberdeen

Fraser Noble Building, AB24 3UE

*Correspondence: juliano.morimoto@abdn.ac.uk

† Present address: Wissenschafskolleg zu Berlin, 10 Wallotstraße, Berlin, Germany.

Short title

Amino acids are unevenly used across proteomes

Abstract

Amino acids are the building blocks of proteins and enzymes which are essential for life. Understanding amino acid usage offers insights into protein function and molecular mechanisms underlying life histories. However, genome-wide patterns of amino acid usage across domains of life remain poorly understood. Here, we analysed the proteomes of 5,590 species across four domains and found that only a few amino acids are consistently the most and least used. This differential usage results in lower amino acid usage diversity at the most and least frequent ranks, creating a ubiquitous inverted U-shape pattern of amino acid diversity and rank which we call an 'edge effect' across proteomes and domains of life. This effect likely stems from protein secondary structural constraints, not the evolutionary chronology of amino acid incorporation into the genetic code, highlighting the functional rather than evolutionary influences on amino acid usage. We also tested other contemporary hypotheses regarding amino acid usage in proteomes and found that amino acid usage varies across life's domains and is only weakly influenced by growth temperature. Our findings reveal a novel and pervasive amino acid usage pattern across genomes with the potential to help us probe deep evolutionary relationships and advance synthetic biology.

Keywords: Genetic code; Structural biology; Environmental responses; Physiology

Supplementary files

Extended Data 1. Amino acid profiles for 5,590 species. (separate Excel).

Extended Data 2. Growth temperature for 296 species of Bacteria and Archaea for which amino acid profiles were estimated. (separate Excel).

Extended Data 3. PDB identification numbers for the structures analysed in this study. (separate Excel).

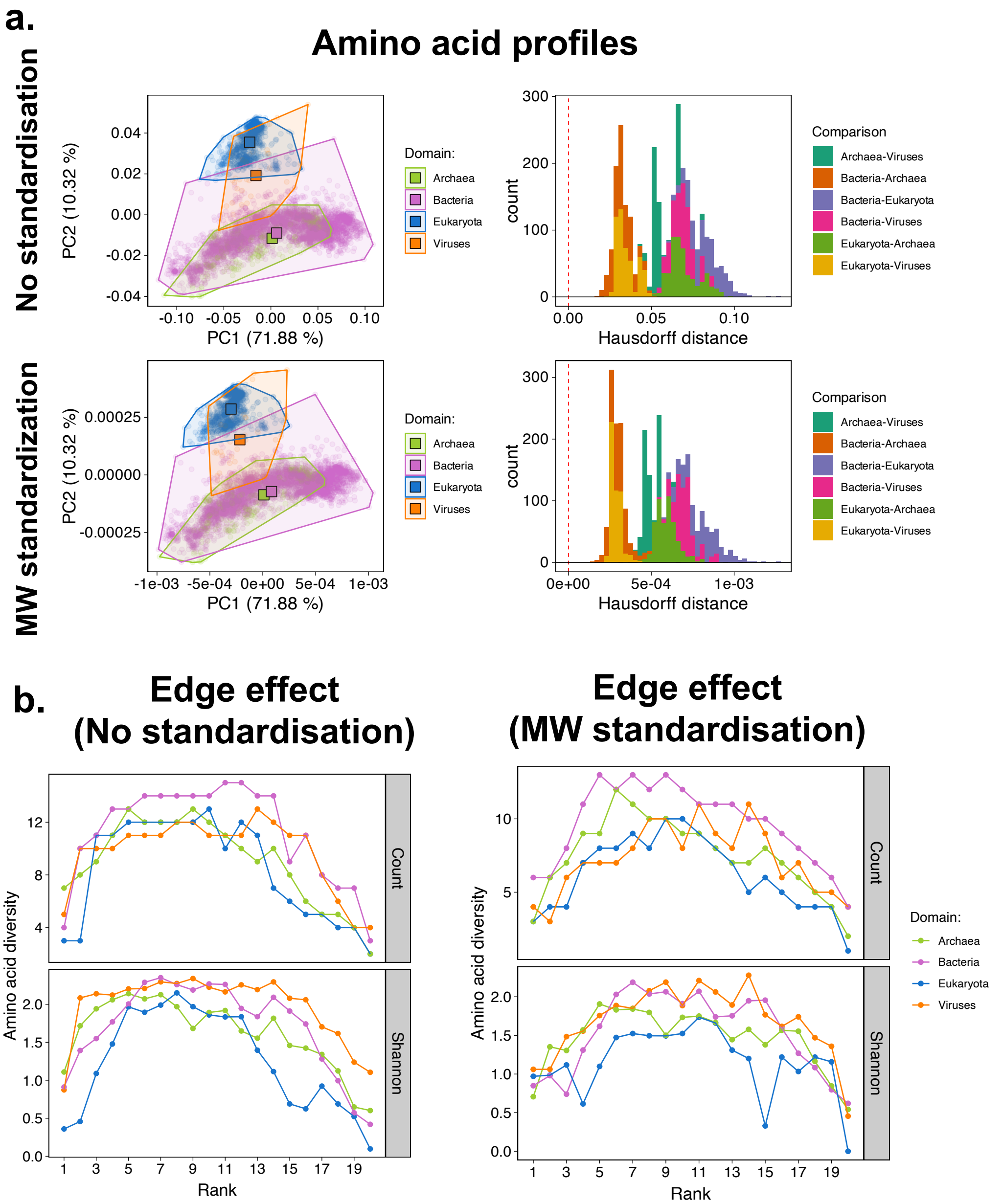

Supplementary File 1. (a) Amino acid profiles differ between domains of life as shown by principal component analysis and the Hausdorff distance in non-standardised (i.e. raw amino acid frequencies in the proteome) and molecular weight-standardised data (i.e. amino acid frequencies divided by amino acid molecular weight). (b) Edge effect can be seen in amino acid profiles for non-standardised and standardised by molecular weight models.

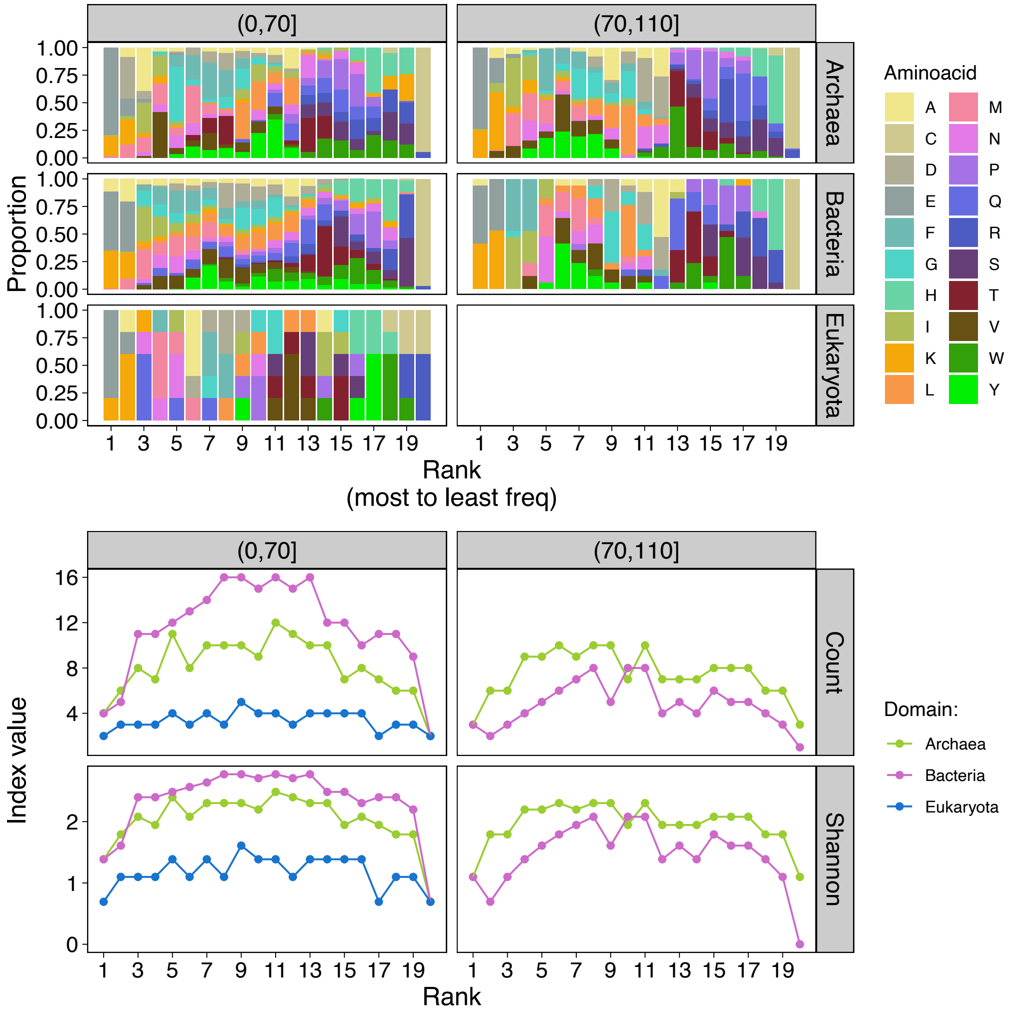

Supplementary File 2. Proportions of amino acid by rank for proteomes of species with optimal growth temperature from 0 to 70ºC and from 70ºC to 110ºC (extremophiles).

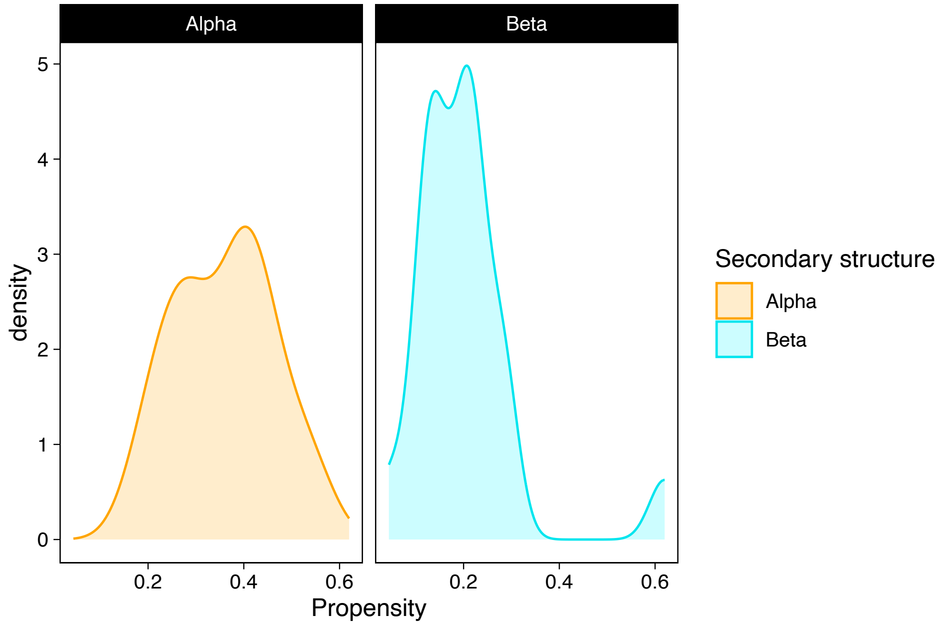

Supplementary File 3. Density plot of the distributions of amino acid propensities for α-helices (symmetric) and β-strands (right-skewed).

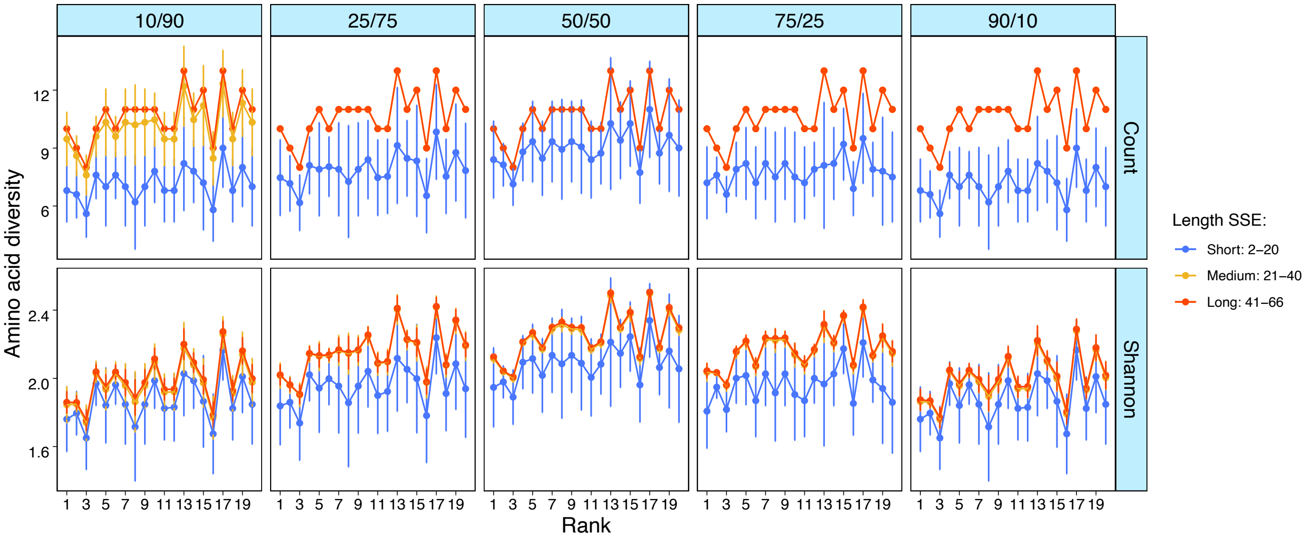

Supplementary File 4. Amino acid diversity by rank, calculated using the Shannon-Wiener index, within simulated proteins. The edge effect disappears in simulations where amino acids were sampled with equal (0.05) probabilities, confirming that differences in secondary structure propensities drive the edge effect.

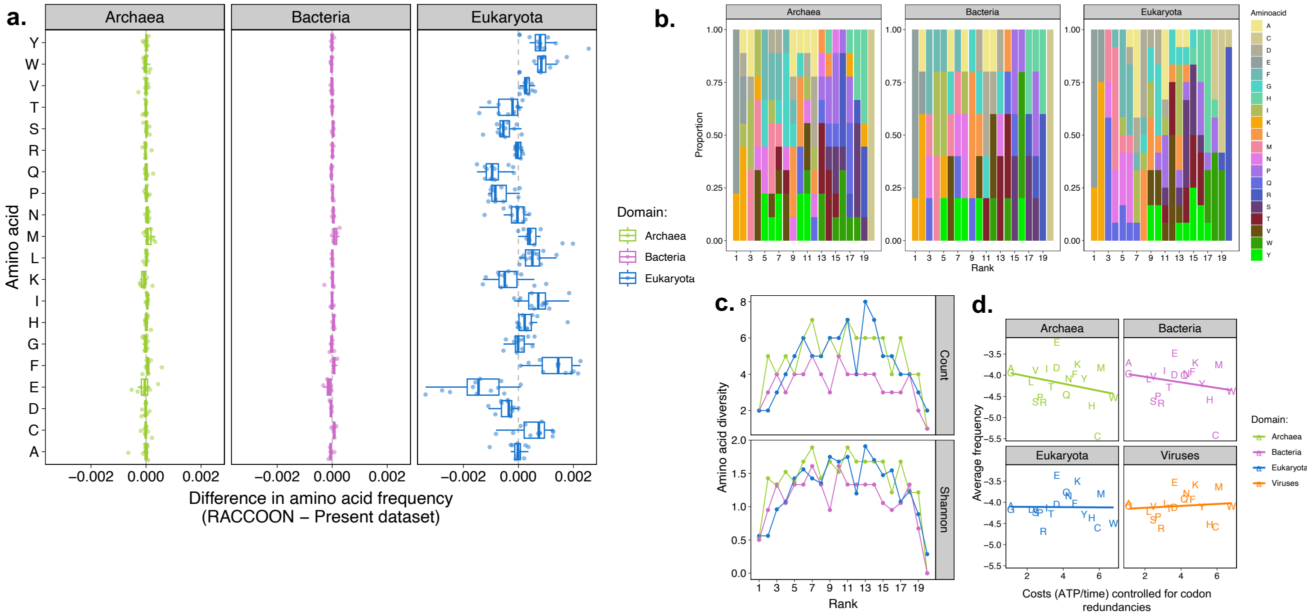

Supplementary File 5. (a) Differences in estimated amino acid frequency between our data and the RACCOON dataset. (b-c) The edge effect described here was also present in the RACCOON dataset. (d) The relationship between amino acid frequency and ATP/time costs from[49].

Supplementary Table 1. Complete outputs for all statistical models reported in this study (separate Excel).
